# Long-Term Neuroprotective Effects of Hydrogen-Rich Water and Memantine in Chronic Radiation-Induced Brain Injury: Behavioral, Histological, and Molecular Insights

**DOI:** 10.3390/antiox14080948

**Published:** 2025-08-01

**Authors:** Kai Xu, Huan Liu, Yinhui Wang, Yushan He, Mengya Liu, Haili Lu, Yuhao Wang, Piye Niu, Xiujun Qin

**Affiliations:** 1 Department of Occupational and Environmental Health, School of Public Health, Capital Medical University, Beijing 100069, China; xukai@mail.ccmu.edu.cn; 2 Beijing Key Laboratory of Environment and Aging, School of Public Health, Capital Medical University, Beijing 100069, China; 3 China Shanxi Key Laboratory of Drug Toxicology and Preclinical Studies for Radiopharmaceutical, Division of Radiation Medicine and Environmental Medicine, China Institute for Radiation Protection, Taiyuan 030006, China; liuhuan@cirp.org.cn (H.L.); qyli0304@gmail.com (Y.W.); hyshan1998@163.com (Y.H.); liumengya728@163.com (M.L.); wyh950103@163.com (Y.W.); 4 CNNC Key Laboratory on Radio-Toxicology and Radiopharmaceutical Preclinical Evaluation, China Institute for Radiation Protection, Taiyuan 030006, China; 5 Collaborative Innovation Center for Molecular Imaging of Precision Medicine, Shanxi Medical University, Taiyuan 030006, China; luhaili1232025@163.com

**Keywords:** hydrogen-rich water, memantine hydrochloride, neuroprotection, radiation-induced brain injury, cognitive impairment, CD44

## Abstract

Hydrogen-rich water (HRW) has shown neuroprotective effects in acute brain injury, but its role in chronic radiation-induced brain injury (RIBI) remains unclear. This study investigated the long-term efficacy of HRW in mitigating cognitive impairment and neuronal damage caused by chronic RIBI. Fifty male Sprague Dawley rats were randomly divided into five groups: control, irradiation (IR), IR with memantine, IR with HRW, and IR with combined treatment. All but the control group received 20 Gy whole-brain X-ray irradiation, followed by daily interventions for 60 days. Behavioral assessments, histopathological analyses, oxidative stress measurements, ^18^F-FDG PET/CT imaging, transcriptomic sequencing, RT-qPCR, Western blot, and serum ELISA were performed. HRW significantly improved anxiety-like behavior, memory, and learning performance compared to the IR group. Histological results revealed that HRW reduced neuronal swelling, degeneration, and loss and enhanced dendritic spine density and neurogenesis. PET/CT imaging showed increased hippocampal glucose uptake in the IR group, which was alleviated by HRW treatment. Transcriptomic and molecular analyses indicated that HRW modulated key genes and proteins, including CD44, CD74, SPP1, and Wnt1, potentially through the MIF, Wnt, and SPP1 signaling pathways. Serum CD44 levels were also lower in treated rats, suggesting its potential as a biomarker for chronic RIBI. These findings demonstrate that HRW can alleviate chronic RIBI by preserving neuronal structure, reducing inflammation, and enhancing neuroplasticity, supporting its potential as a therapeutic strategy for radiation-induced cognitive impairment.

## 1. Introduction

Radiotherapy remains one of the most effective treatment modalities for both primary brain tumors and brain metastases [1]. Each year, hundreds of thousands of patients worldwide undergo brain irradiation, with over 100,000 surviving beyond six months annually in the United States alone [2]. Despite its therapeutic efficacy, brain irradiation often results in progressive cognitive impairment, affecting 50–90% of long-term survivors [2]. These impairments profoundly diminish patients’ quality of life, manifesting as memory loss, attention deficits, and executive dysfunction, which pose significant barriers to rehabilitation and independence [3].

Chronic radiation-induced brain injury (RIBI) is the predominant form of late-delayed neurotoxicity, typically emerging months after radiotherapy. It is characterized by cortical dysfunction, hippocampal degeneration, neuroinflammation, and blood–brain barrier (BBB) disruption. Importantly, chronic RIBI is not only irreversible but also challenging to diagnose [4]. Current clinical interventions, such as corticosteroids and bevacizumab, provide only limited symptomatic relief and are associated with significant side effects [5,6]. Therefore, identifying safe and effective neuroprotective strategies remains an urgent unmet clinical need.

Mechanistically, RIBI is thought to arise from a cascade of events initiated by ionizing radiation-induced oxidative stress and vascular damage [7,8]. Reactive oxygen species (ROS) disrupt endothelial cells, leading to increased BBB permeability and the release of proinflammatory cytokines. These factors activate resident microglia and astrocytes, promoting a neuroinflammatory microenvironment that exacerbates neuronal injury and synaptic degeneration [9,10]. Chronic neuroinflammation, coupled with impaired neurogenesis and synaptic loss, ultimately contributes to the cognitive impairments characteristic of late-delayed RIBI. Understanding this temporal and molecular progression is critical to identifying intervention windows and therapeutic targets.

Memantine is a well-established neuroprotective agent approved for moderate-to-severe Alzheimer’s disease [11]. As a low-to-moderate affinity, voltage-dependent, non-competitive antagonist of the NMDA receptor, memantine prevents excitotoxic neuronal injury without interfering with normal synaptic transmission. Preclinical and clinical evidence suggests that memantine not only attenuates glutamate-mediated neurotoxicity but may also reduce oxidative stress, neuroinflammation, and hippocampal apoptosis, thereby offering therapeutic benefits in various CNS injury models. Notably, recent clinical trials have expanded its potential application to radiation-induced cognitive dysfunction, supporting its relevance in radiation-related neuroprotection [12,13,14].

Molecular hydrogen (H_2_) has emerged as a selective antioxidant and anti-inflammatory agent with the ability to rapidly diffuse across cellular membranes and the blood–brain barrier. It can be administered through various routes, including inhalation of hydrogen gas, oral intake of hydrogen-rich water, and injection of hydrogen-dissolved saline [15]. Pharmacokinetic studies in both humans and animal models have demonstrated that molecular hydrogen (H_2_) is rapidly absorbed and distributed following oral or gavage administration of hydrogen-rich water [16]. Due to its small molecular size and non-polar nature, H_2_ readily diffuses across biological membranes, including the blood–brain barrier [17]. In human subjects, drinking 500 mL of saturated HRW led to detectable levels of H_2_ in blood and exhaled breath within 10 min, with concentrations returning to baseline within approximately one hour [18]. Around 59% of the ingested hydrogen is eliminated via respiration, ~0.1% is lost through transdermal diffusion, and approximately 40% is retained and utilized in tissues [18]. In rodents, elevated H_2_ levels in both blood and brain tissue have been detected within minutes after administration [17]. Unlike conventional antioxidants, H_2_ selectively scavenges highly cytotoxic species such as hydroxyl radicals (^•^OH) and peroxynitrite (ONOO^−^), while preserving physiological reactive oxygen species (ROS) involved in normal cellular signaling [16]. Experimental studies have demonstrated that hydrogen therapy mitigates neuronal injury by modulating redox homeostasis, inhibiting glial activation, and preserving mitochondrial function. Notably, H_2_ has shown neuroprotective efficacy across a broad range of central nervous system (CNS) disorders, including ischemia–reperfusion injury, neurodegenerative diseases, and radiation-induced brain injury [19].

Our previous research focused on the acute phase of RIBI, revealing that both memantine—a non-competitive NMDA receptor antagonist—and hydrogen-rich water (HRW)—a selective antioxidant—can attenuate neuronal injury by suppressing pyroptosis through the NLRP3/NLRC4/Caspase-1 inflammasome signaling pathway [20]. However, the pathophysiology of chronic RIBI is likely more complex and may involve a mechanistic shift from early-phase inflammasome activation to long-term neurodegenerative processes. However, it remains unclear whether the same mechanisms underlie chronic RIBI, which may involve distinct pathological pathways such as extracellular matrix remodeling, glial reactivity, and immune-mediated neurodegeneration.

To address these limitations, the present study investigates the long-term neuroprotective effects of HRW and memantine in a chronic RIBI mouse model. We implemented a modified irradiation protocol—with adjusted dose and targeted shielding—to better simulate the chronic pathological context. Using an integrated assessment approach encompassing behavioral tests, PET/CT imaging, histological analysis, and transcriptomic profiling, we sought to determine whether HRW confers neuroprotection through pyroptosis-independent mechanisms. Particular focus was placed on identifying novel molecular pathways—including CD44/MIF, SPP1, and Wnt signaling—that may mediate neuroinflammation, neurogenesis, and synaptic preservation in the chronic phase. This study offers critical insights into the temporal dynamics of RIBI and provides a foundation for the development of non-toxic, translationally relevant interventions to preserve cognitive function in patients receiving brain radiotherapy.

## 2. Materials and Methods

### 2.1. Animals and Ethics Statement

Fifty male Sprague Dawley (SD) rats, 6 weeks old and weighing 210–240 g, were obtained from Beijing Vital River Laboratory Animal Technology Co., Ltd., Beijing, China (Certificate No.: 110011230100562657). Rats were housed at the Animal Experiment Center of the China Institute for Radiation Protection [SYXK (Jin) 2018-0005] under controlled temperature, humidity, and light conditions, with a 12 h light/dark cycle (light: 7:00–19:00; dark: 19:00–7:00). All animals had free access to food and water. After a 9-day acclimatization period, the rats were randomized into experimental groups and received interventions for two days prior to irradiation. At the time of irradiation, the rats weighed between 290 and 340 g. All animal procedures were approved by the Ethics Committee for Animal Experimentation of the China Institute for Radiation Protection (Approval No.: CIRP-IACUC-(R)2022007) on 17 May 2022 and were conducted in accordance with relevant institutional and national ethical guidelines.

### 2.2. Animal Grouping and Treatment

To ensure balanced body weight distribution across groups, rats were first stratified into weight-based blocks. Within each block, animals were randomly assigned to one of five groups (*n* = 10 per group) using random numbers generated by Stata software 17.0. The groups were as follows: control (no treatment), irradiation only (IR), memantine treatment (IR + memantine/MEM), hydrogen-rich water treatment (IR + HRW), and joint treatment (IR + joint). Each animal received a daily intragastric gavage starting 2 days prior to irradiation and continuing until 60 days post-irradiation, with the gavage on the day of irradiation administered 30 min before exposure. The control and IR groups received pure water, while the memantine dosage for the IR + memantine group was based on the clinical recommendation of 20 mg/day, adjusted for rats using Meeh’s formula for body surface area, approximately 2.5 mg/(kg·day). The IR + memantine group received the calculated dose of memantine, prepared by dissolving the drug in pure water to achieve a concentration of 0.125 mg/mL, and administered at 2.5 mg/kg with a dosing volume of 20 mL/kg. The IR + HRW group received hydrogen-rich water with a concentration ≥1000 ppb (or 0.5 mM), which was prepared using the ZFFQ-500 hydrogen-rich water generator (Shanxi Zhongfu Nuclear Instrument Co., Ltd., CIRNIC, Shijiazhuang, China). The hydrogen gas concentration in the hydrogen-rich water was measured using the ENH-1000 portable dissolved hydrogen meter (Trustlex, Suita, Japan). The joint group received memantine (2.5 mg/kg) dissolved in hydrogen-rich water. Memantine hydrochloride (Batch No. Q63220901) was obtained from CSPC OUYI Pharmaceutical Co., Ltd. (Shijiazhuang, China). The gavage volume was standardized at 20 mL/kg for all groups.

### 2.3. Animal Irradiation Conditions

To accurately target the rat brain, cranial imaging was performed to determine the precise irradiation area, extending from the supraorbital ridge to the posterior edge of the ears (Figure S1a). All rats were anesthetized with an intraperitoneal injection of sodium pentobarbital (50 mg/kg) and positioned prone with their backs facing the machine head. Lead shields were used to protect the oral cavity and eyes (Figure S1b). The irradiation was conducted using a 6 MeV Elekta Synergy linear accelerator (Elekta, Crawley, UK) with an X-ray dose rate of 300 MU/min. All groups except the control group received whole-brain irradiation at a total dose of 20 Gy.

### 2.4. Timeline and Data Collection

The body weight and food intake of the rats were measured at three time points: 3 days prior to grouping (−3d), 1 day post-irradiation, and weekly thereafter until the 8th week post-irradiation. Behavioral experiments were conducted on days 30 and 60 post-irradiation. Bromodeoxyuridine (BrdU) was administered intraperitoneally at a dosage of 50 mg/kg daily for 7 consecutive days (from days 36 to 42 post-irradiation). Three rats per group were used for BrdU + NeuN fluorescence double-labeling analysis.

Data acquisition via ^18^F-fluorodeoxyglucose positron emission tomography/computed tomography (^18^F-FDG PET/CT) was performed on days 35 and 71–72 post-irradiation. On day 74 post-irradiation, rats were anesthetized with intraperitoneal injection of sodium pentobarbital, followed by blood collection via the abdominal aorta. The whole brain was then carefully extracted.

**Figure 1:**
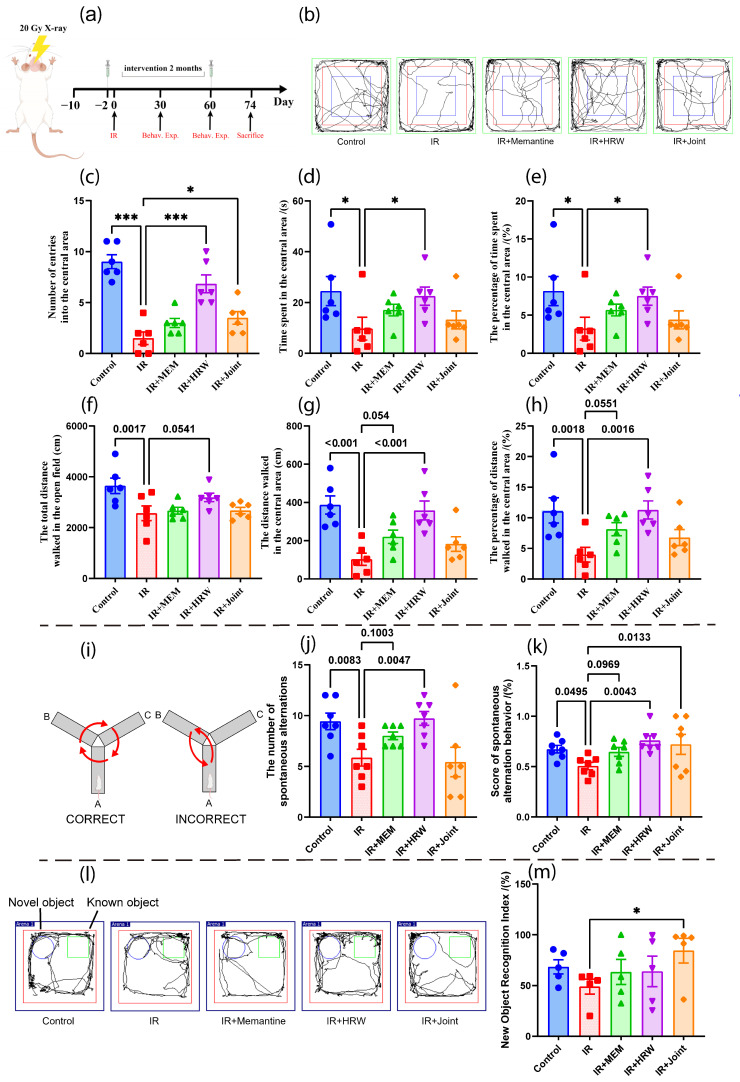
Experimental timeline and behavioral tests on day 30 post-irradiation. (**a**) Experimental timeline. (**b**) Trajectories of rats in the open field on day 30 post-irradiation. The blue area indicates the central zone. (**c**–**h**) Behavioral indices of rats on day 30 post-irradiation in the open field (*n* = 6): (**c**) number of entries into the central area, (**d**) time spent in the central area, (**e**) percentage of time spent in the central area, (**f**) total walking distance, (**g**) walking distance in the central area, and (**h**) percentage of central area walking distance relative to total distance. (**i**) Schematic of the Y-Maze spontaneous alternation test. (**j**) Number of alternations in the Y-Maze spontaneous alternation test on day 30 post-irradiation (*n* = 5). (**k**) Spontaneous alternation score in the Y-Maze spontaneous alternation test on day 30 post-irradiation. (**l**) Trajectory of rats in the Novel Object Recognition Test on day 30 post-irradiation. (**m**) Novel object recognition index. (* *p* < 0.05, *** *p* < 0.001, compared with the IR group.).

For subsequent analyses, five rats per group were allocated to pathological and histological examinations, while another five rats per group were used for biochemical analyses after isolating the hippocampus and cerebral cortex. The timeline of these experimental procedures is illustrated in Figure 1a.

### 2.5. Behavioral Experiments

Behavioral experiments were conducted on days 30 and 60 post-irradiation to evaluate cognitive and emotional changes. On day 30, the Open Field Test (OFT), Y-Maze spontaneous alternation test, and Novel Object Recognition Test (NORT) were performed. The OFT was conducted in a 100 cm × 100 cm × 40 cm arena to assess anxiety-like behaviors and spontaneous activity, recording the time spent in the central area and the total distance traveled over 5 min [21].

The Y-Maze spontaneous alternation behavior test is widely used to assess spatial working memory, exploration tendency, and cognitive deficits in rodents [22]. The apparatus consists of three arms arranged at 120° angles, labeled A, B, and C, forming a “Y” shape. Each arm measures 60 cm in length, 10 cm in width, and 18 cm in height. The rat is placed at the center of the maze and allowed to explore freely for 5 min, with behavior recorded by a video system, as shown in the schematic of the Y-Maze spontaneous alternation test (Figure 1i). The following parameters are recorded. (1) Total number of entries: the total number of times the rat enters an arm, defined as the rat entering with all four paws. (2) Alternation: a sequence where the rat enters all three arms without repetition (e.g., A → B → C). (3) Maximum alternations: calculated as the total number of entries minus 2 (Total entries − 2). (4) Spontaneous alternation score: calculated as (Total alternations/Maximum alternations) × 100%. The data are analyzed to assess the animal’s spatial working memory, with higher alternation scores indicating better memory and the ability to recall previous choices.

The Novel Object Recognition (NOR) Test is a non-reward-based cognitive memory task used to assess memory function in rodents [23]. The apparatus consists of a 40 × 40 cm base and a 45 cm high box with two identical rectangular objects and one cylindrical object, all with the same height. The experiment is conducted over two days. On the first day, two identical rectangular objects are placed symmetrically in the box, with the rat placed facing away from them for 10 min. On the second day, one rectangular object is replaced with a cylindrical object, and the rat’s behavior is recorded for 5 min. If the rat’s cognitive function is normal, it will spend more time exploring the new object. The Recognition Index (RI) is calculated as follows: RI = Time exploring new object/(Time exploring new object + Time exploring old object) ×100%

On day 60, the OFT and Y-Maze alternation test were repeated to assess consistency. The Morris Water Maze (MWM) [24] was used to evaluate spatial learning and memory over 8 days, including 5 days of training to locate a hidden platform, followed by probe trials on days 6 and 8 to assess memory consolidation, with the rat entry positions during the MWM trials shown in Table 1. To prevent any interference from the Y-Maze alternation test, the Y-Maze novel arm exploration test was conducted after the water maze trials. The novel arm exploration test uses the same apparatus as the Y-Maze spontaneous alternation test and is designed to assess memory and cognitive function in rats. The experiment has two phases: In the first phase (acquisition), one arm of the Y-Maze is closed, and the rat explores the remaining two arms for 3 min. Five hours later, in the second phase (recall), all arms are open, and the rat explores freely for 3 min (Figure S2b). The time and distance explored in each arm are recorded. If the rat’s memory is impaired, it will explore the new arm less. The following parameters are analyzed: the number of entries into the novel arm, the latency to first enter the novel arm, and the percentage of time spent in the novel arm.

To prevent interference from odors or residues, all equipment was thoroughly cleaned with alcohol and paper towels between tests. Behavioral data were recorded and analyzed using SMART Panlab 3.0 software.

### 2.6. Histopathological Evaluation

Histopathological evaluation included H&E staining, Nissl staining, Golgi staining, BrdU + NeuN double immunofluorescence labeling, and TEM. Brain tissues were fixed in 4% paraformaldehyde for 48 h, followed by dehydration, clearing, paraffin embedding, and sectioning. H&E staining was used to evaluate tissue lesions under a microscope, with severity categorized from “+” (mild) to “++++” (severe). BrdU+NeuN double immunofluorescence labeling was conducted on paraffin sections following antigen retrieval, after intraperitoneal BrdU administration (50 mg/kg daily for 7 days starting on day 36 post-irradiation). Sections were incubated with BrdU and NeuN antibodies, and DAPI was used to stain cell nuclei. Newborn neurons (BrdU+ labeled) and mature neurons (NeuN+ labeled) were visualized under a fluorescence microscope. Hippocampal tissues for TEM were fixed in glutaraldehyde, treated with osmium tetroxide, dehydrated, and embedded. Ultra-thin sections (60–80 nm) were stained with uranyl acetate and lead citrate, and ultrastructural changes were observed under TEM. For Golgi staining, brain tissues were treated with Golgi stain fixative for 14 days, sectioned, and developed. Dendritic structures were visualized under a microscope, and dendritic spine density was analyzed using ImageJ version 1.52i (National Institutes of Health, Bethesda, MD, USA). Nissl staining involved deparaffinization of sections in xylene and ethanol, followed by staining and differentiation with 0.1% glacial acetic acid. Neurons in the hippocampal CA1 region were counted under a microscope.

### 2.7. ^18^F-FDG PET/CT Static Imaging and Data Reconstruction Analysis

PET/CT imaging (Inveon PET/CT, Siemens, Munich, Germany) was performed on days 35 and 70 post-exposure to assess changes in brain glucose uptake. On day 35, only the control and model groups were imaged to evaluate the effects of ionizing radiation on brain glucose metabolism. ^18^F-FDG was provided by the Department of Nuclear Medicine at the First Hospital of Shanxi Medical University. Rats were fasted for 4 h prior to imaging, with free access to water. Anesthesia was induced and maintained with 2% isoflurane throughout the procedure. Each rat received a tail vein injection of ^18^F-FDG at an activity of 7.0 ± 0.5 MBq in a 0.6 mL volume, with net activity calculated by subtracting residual syringe activity. Static PET imaging was conducted at 30 and 60 min post-injection, with a 10 min acquisition time, followed by a 40 s CT scan. During imaging, continuous gas anesthesia was maintained, and respiratory and heart rates were monitored to adjust gas flow as needed. Imaging data were reconstructed and processed using PMOD software (version 4.006, PMOD Technologies LLC, Zurich, Switzerland), aligning micro-PET/CT data with MRI images of rat brains. ^18^F-FDG uptake in the bilateral hippocampi was quantified, and intergroup differences were analyzed for statistical significance.

### 2.8. Oxidative Stress Biomarker Assessment

Brain tissue samples were homogenized on ice and processed following the kit instructions to measure oxidative stress biomarkers, including superoxide dismutase (SOD), reduced glutathione (GSH), and hydroxyl radicals (OH^•^), in the cerebral cortex. Absorbance was measured using a microplate reader (Thermo, Waltham, MA, USA). SOD activity was determined with the SOD assay kit (Beyotime, Shanghai, China, S0101S), GSH levels with the GSH assay kit (Nanjing Jiancheng Bioengineering Institute, Nanjing, China, A006-2-1), and OH^•^ concentrations with the hydroxyl radical assay kit (Nanjing Jiancheng Bioengineering Institute, Nanjing, China,, A018-1-1). Protein concentrations were quantified using the BCA Protein Assay Kit (Thermo Scientific, Waltham, MA, USA, 23227) to normalize the oxidative stress marker levels.

### 2.9. Transcriptomic Sequencing of Hippocampal Tissue

To investigate the mechanisms by which memantine and hydrogen-rich water ameliorate radiation-induced cognitive impairment, transcriptomic sequencing was performed on hippocampal tissue samples from the control, IR, and HRW groups, with 4 samples per group. Differentially expressed genes (DEGs) were identified using the criteria of *p* < 0.05 and |log fold change| > 1 and visualized with volcano plots. Functional enrichment analyses, including KEGG and GO pathway analyses, were conducted to identify biological processes and pathways associated with the DEGs. Gene set enrichment analysis (GSEA) was performed using the clusterProfiler package in R to determine whether predefined gene sets exhibited statistically significant and consistent differences between two biological states. The analysis utilized the C2: curated gene sets and C5: GO gene sets from MSigDB (https://www.gsea-msigdb.org/gsea/msigdb, accessed on 11 December 2023). Pathways were considered significantly enriched if |NES| > 1, NOM *p*-value < 0.05, and FDR < 0.25. Protein-protein interaction (PPI) networks were constructed using the STING database and visualized with Cytoscape software version 3.10.2 (The Cytoscape Consortium, San Diego, CA, USA). Genes with high connectivity (degree) in the PPI network were identified as candidate key genes for further validation. Subsequent experiments, including RT-qPCR, Western blot, and ELISA, were performed to validate these candidate genes and associated pathways.

### 2.10. Real-Time Quantitative PCR (RT-qPCR) Analysis

Hippocampal tissue samples (40–50 mg) were homogenized in 1 mL of TRIzon Reagent (Invitrogen) using an electric glass homogenizer. Subsequently, 200 µL of chloroform was added, and the mixture was incubated at room temperature for 3–5 min. The samples were centrifuged at 12,000 rpm for 15 min to separate the phases. The aqueous layer was collected and mixed with 1 mL of isopropanol, followed by a 10 min incubation at room temperature. After centrifugation at 12,000 rpm for 10 min, the supernatant was discarded, and the precipitate was washed with 1 mL of 75% ethanol. The samples were centrifuged again at 12,000 rpm for 5 min, and the ethanol was discarded. The pellet was air-dried for 5–10 min until a gel-like product was obtained. Total RNA was dissolved in sterile, enzyme-free water, and its concentration and purity were measured using a NanoDrop spectrophotometer (Thermo Fisher Scientific, Waltham, MA, USA). The extracted RNA was stored at −80 °C until use. Prior to reverse transcription, RNA concentrations were normalized across samples to ensure consistency. Genomic DNA was removed, and RNA was reverse-transcribed into single-stranded cDNA using the RT III Reverse Transcription Kit (TOYOBO, Osaka, Japan). Real-time quantitative PCR (RT-qPCR) was performed using the SYBR Green method, with GAPDH serving as the internal control gene. The relative expression levels of target genes, including *Cd44*, *Cd74*, *Cd3e*, *RT1-Ba*, *RT1-Da*, *Spp1*, *Adipoq*, and *Kif18a*, were calculated using the 2^−ΔΔCt^ method. Primer sequences are listed in Table 2, with primers synthesized by Shanghai Sangon Biotech Co., Ltd, Shanghai, China. The PCR amplification protocol consisted of an initial cycle at 50 °C for 2 min and 95 °C for 45 s, followed by 40–55 cycles at 95 °C for 15 s, 55–60 °C for 15 s, and 72 °C for 45 s. A melt curve analysis was performed at the end of the reaction to confirm the specificity of the amplified products.

### 2.11. Western Blotting Analysis

Hippocampal tissue samples (20–30 mg) were cut into small fragments and homogenized on ice with 200 µL of RIPA lysis buffer (New Cell & Molecular Biotech, Suzhou, China, WB3100) containing protease and phosphatase inhibitor cocktails (New Cell & Molecular Biotech, P002). Homogenized samples were incubated at room temperature for 30 min and centrifuged at 14,000 rpm for 15 min to collect the supernatant. Protein concentrations were determined using the BCA method, normalized, and denatured at 95 °C. Proteins were separated via SDS-PAGE and transferred to NC or PVDF membranes. Membranes were blocked with 5% nonfat dry milk and incubated overnight at 4 °C with primary antibodies. The next day, membranes were washed three times with TBST (10 min per wash) at room temperature and incubated with HRP-conjugated secondary antibodies (1: 5000 dilution) at 37 °C for 1 h. After washing three more times with TBST, protein bands were visualized using an enhanced chemiluminescent (ECL) substrate. Images were captured using an imaging analyzer, and semi-quantitative analysis was performed using ImageJ version 1.52i (National Institutes of Health, Bethesda, MD, USA). Protein expression levels were normalized to the grayscale values of internal control bands. The following primary antibodies were used: CD44 antibody (Wan Lei Biotechnology Co., Ltd., Shenyang, China; WL03531), CD74 antibody (Huamei Biotechnology Co., Ltd., Wuhan, China; CSB-PA004956LA01RA), SPP1 antibody (Huamei Biotechnology Co., Ltd., Wuhan, China; CSB-PA827743), and Wnt1 antibody (Wan Lei Biotechnology Co., Ltd., Shenyang, China; WL05209).

### 2.12. Rat Serum ELISA Detection

Serum levels of CD44 (Nanjing SenBeiJia Biological Technology Co., Ltd., Nanjing, China; SBJ-R0237) and AdipoQ (Shanghai Enzyme-linked Biotechnology Co., Ltd., Shanghai, China; ml002882) were quantified using enzyme-linked immunosorbent assay (ELISA). The protocol was conducted according to the manufacturer’s instructions, ensuring the accurate determination of assay detection limits and the optimal dilution factor for serum samples. All reagents were prepared as instructed, and serum samples were diluted appropriately based on the detection range. Samples and standards were added to the microplate wells, followed by incubation and washing steps as specified in the kit protocols. The final reaction was developed using the provided substrate solution, and absorbance values were measured using a microplate reader. The concentrations of CD44 and AdipoQ were calculated by comparing the absorbance values of samples to the standard curve.

### 2.13. Statistical Analysis

Statistical analyses and data visualization were performed using GraphPad Prism 9.0 (San Diego, CA, USA). Group differences were evaluated using one-way analysis of variance (ANOVA) for comparisons involving two or more groups, followed by uncorrected least significant difference (LSD) post hoc tests for pairwise comparisons. For datasets that did not meet the assumptions of normality or homogeneity of variance, non-parametric tests were applied. Results are expressed as mean ± standard error of the mean (SEM), and statistical significance was defined as *p* < 0.05.

## 3. Results

### 3.1. General Conditions of Animals

All rats survived the 74-day post-irradiation period. Statistical analysis revealed significant differences in body weight between the control group and the irradiated groups starting from day 7 post-irradiation. However, there were no significant differences in body weight among the IR group and the various intervention groups. The changes in body weight across the groups are shown in Figure S2a.

Food intake varied significantly between the irradiated groups and the control group. In all irradiated groups, food intake sharply decreased to nearly zero within the first week post-irradiation. By day 10, food intake gradually began to recover, surpassing that of the control group by day 15. From day 30 post-irradiation until dissection, food intake in the irradiated groups remained consistently lower than that in the control group. Similar to body weight, there were no significant differences in food intake among the IR group and the various intervention groups, as shown in Figure S2b.

### 3.2. Behavioral Results

#### 3.2.1. Open Field Test on Day 30 Post-Irradiation

The trajectories of rats in the open field are shown in Figure 1b. Compared to the IR group, all intervention groups demonstrated varying degrees of behavioral recovery. The control group and the IR + HRW group showed significantly higher values in the number of entries into the central area, time spent in the central area, percentage of time in the central area, walking distance in the central area, and the percentage of central area distance relative to total distance (*p* < 0.05), as shown in Figure 1c–h.

#### 3.2.2. Y-Maze Spontaneous Alternation Test on Day 31 Post-Irradiation

In the Y-Maze spontaneous alternation test, both the number of alternations and the spontaneous alternation scores in the Control and IR + HRW groups were significantly higher than those in the IR group. Compared to the IR group, improvements in these parameters were also observed in the IR + memantine and IR + joint groups. Notably, the spontaneous alternation score in the IR + joint group was significantly higher than that in the IR group (Figure 1g,h).

#### 3.2.3. Novel Object Recognition Test on Days 32–33 Post-Irradiation

In the novel object recognition test, the control group and all intervention groups exhibited higher novel object recognition indices compared to the IR group. However, only the IR + Joint group demonstrated a statistically significant improvement in the novel object recognition index relative to the IR group (*p* < 0.05), as shown in Figure 1l,h.

#### 3.2.4. Open Field Test on Day 60 Post-Irradiation

**Figure 2:**
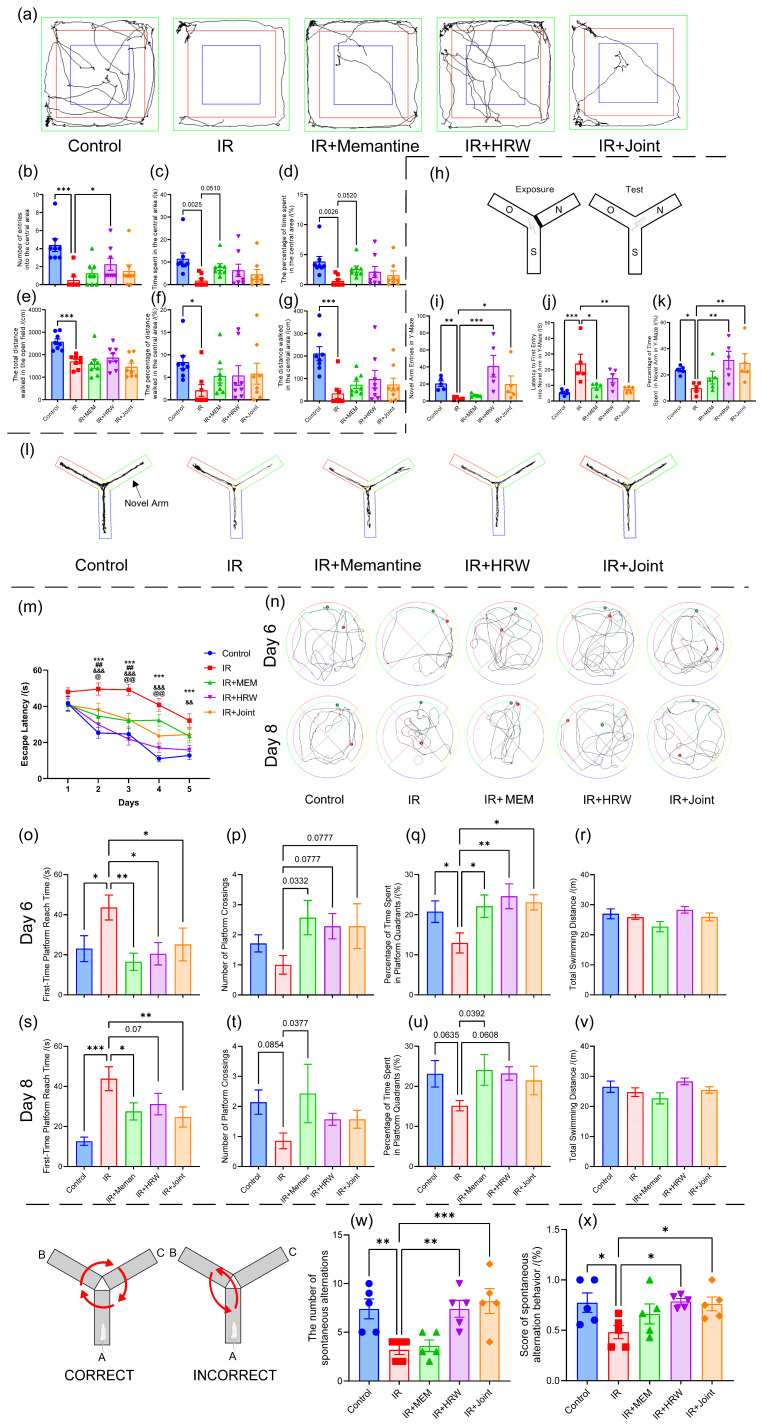
Behavioral tests on days 60–73 post-irradiation. (**a**–**g**) Open field test on day 60 post-irradiation (*n* = 8 per group): (**a**) trajectories of rats in the open field on day 60 post-irradiation for each group; (**b**) number of entries into the central area; (**c**) time spent in the central area (seconds); (**d**) percentage of time spent in the central area (%); (**e**) total distance walked in the open field (cm); (**f**) percentage of distance walked in the central area (%); (**g**) distance walked in the central area (cm). (**h**–**i**) Y-maze novel arm exploration test on day 61 (*n* = 5): (**h**) schematic of the Y-maze novel arm exploration test; (**i**) number of entries into the novel arm; (**j**) latency to first entry into the novel arm in the Y-maze (seconds); (**k**) percentage of time spent in the novel arm in the Y-maze (%); (**l**) trajectories of novel arm exploration in the Y-maze(The green arm represents the Novel Arm, the blue arm represents the Start Arm, and the red arm represents the Other Arm (Familiar Arm)). (**m**–**v**) Water maze test, *n* = 7 per group: (**m**) escape latency trend in the water maze (*: Control group vs. IR group; #: Memantine group vs. IR group; &: HRW group vs. IR group; @: Combined treatment group vs. IR group; *** *p* < 0.001, Control group vs. IR group; ## *p* < 0.01, Memantine group vs. IR group; && *p* < 0.01, HRW group vs. IR group; &&& *p* < 0.001, HRW group vs. IR group; @ *p* < 0.05, Joint group vs. IR group; @@ *p* < 0.01, Joint group vs. IR group); (**n**–**v**) spatial exploration in the water maze; (**n**) trajectories of spatial exploration in the water maze; (**o**) first-time platform reach time in the water maze on day 6; (**p**) number of platform crossings on day 6; (**q**) percentage of time spent in platform quadrants on day 6; (**r**) total swimming distance on day 6; (**s**) first-time platform reach time on day 8; (**t**) number of platform crossings on day 8; (**u**) percentage of time spent in platform quadrants on day 8; (**v**) total swimming distance on day 8. (**w**,**x**) Y-maze spontaneous alternation test at day 73 post-irradiation: (**w**) number of spontaneous alternations; (**x**) score of spontaneous alternation behavior (%). (* *p* < 0.05, ** *p* < 0.01, *** *p* < 0.001, compared with the IR group).

Compared to the control group, the IR group displayed significantly impaired performance in all open field test metrics (*p* < 0.05). The drug intervention groups showed improvements over the IR group, with the number of entries into the central area in the IR + HRW group reaching statistical significance (*p* < 0.05), as shown in Figure 2a–g.

#### 3.2.5. Y-Maze Novel Arm Exploration Test on Day 61 Post-Irradiation

The trajectory maps from the Y-maze novel arm exploration test revealed reduced exploration of the novel arm in the IR group, as shown in Figure 2l. The number of entries into the novel arm significantly increased in the control, IR + HRW, and IR + joint groups compared to the IR group (*p* < 0.05, Figure 2i). The latency to first enter the novel arm was significantly shorter in the control, IR + memantine, and IR + joint groups compared to the IR group (*p* < 0.05, Figure 2j). The percentage of time spent in the novel arm was significantly longer in the control, IR + HRW, and IR + joint groups compared to the IR group (*p* < 0.05, Figure 2k).

#### 3.2.6. Morris Water Maze Test on Days 63 to 70 Post-Irradiation

During the acquisition phase over the first 5 days, all groups showed a progressive reduction in escape latency with training (F_(time)_ = 30.03, *p* < 0.001). ANOVA revealed significant group differences in escape latency (F_(group)_ = 31.24, *p* < 0.001). On day 1, no significant group differences were observed (*p* > 0.05). However, from day 2 onward, the control and IR + HRW groups had significantly shorter escape latencies compared to the IR group (*p* < 0.05). The IR + memantine group exhibited significant improvements on days 2–3, while the IR + Joint group showed improvements on days 2–4 compared to the IR group (*p* < 0.05, Figure 2m).

Typical trajectories for spatial exploration are shown in Figure 2n. The IR group exhibited fewer crossings of the virtual platform and disorganized trajectories. On days 6 and 8 of the spatial exploration tests, no significant differences were observed in the total swimming distance among groups (Figure 2r,v). However, the Control group and all intervention groups displayed superior performance compared to the IR group in escape latency, platform crossings, and percentage of time in the platform quadrant.

On day 6, the time to the first platform entry was significantly shorter in the control and intervention groups compared to the IR group (*p* < 0.05, Figure 2o). Compared to the IR group, the number of platform crossings in the control group and each intervention group increased, with the IR + memantine group reaching a significant level (Figure 2p). Additionally, the percentage of time spent in the platform quadrant was significantly higher (*p* < 0.05, Figure 2q). On day 8, the control, IR + memantine, and IR + joint groups had significantly shorter times to first platform entry compared to the IR group (*p* < 0.05), while the IR + HRW group showed a marginal trend (*p* = 0.07, Figure 2s). The number of platform crossings and the percentage of time spent in the platform quadrant showed some recovery in each intervention group compared to the IR group (Figure 2t,u).

#### 3.2.7. Y-Maze Spontaneous Alternation Test on Day 73 Post-Irradiation

In the Y-maze spontaneous alternation test, the control, IR + HRW, and IR + joint groups exhibited significantly higher alternation counts and spontaneous alternation behavior scores compared to the IR group (*p* < 0.05, Figure 2w,x).

### 3.3. Histopathological Findings

#### 3.3.1. HE Staining

**Figure 3:**
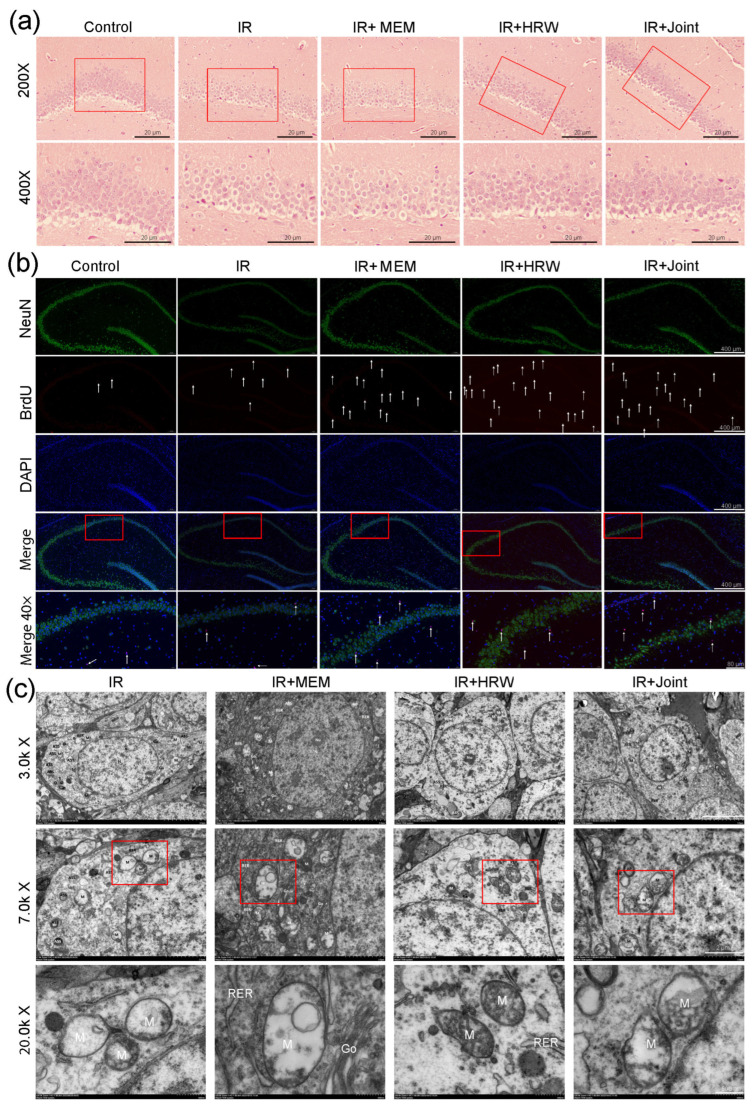
Effects of chronic RIBI intervention on hippocampal structure. (**a**) HE staining of the hippocampal DG region after chronic RIBI intervention. (**b**) BrdU + NeuN immunofluorescence for hippocampal neurogenesis (The arrows and red dots indicate newborn neurons). (**c**) Transmission electron microscopy of hippocampal ultrastructure. Note: Nucleus (N), nucleolus (Nu), mitochondria (M), rough endoplasmic reticulum (RER), Golgi apparatus (Go), autophagolysosome (ASS).

Hematoxylin and eosin (HE) staining of hippocampal brain sections following ionizing radiation (*n* = 3) revealed a disorganized arrangement of neuronal cells in the IR group, accompanied by noticeable swelling and degeneration of neurons in the dentate gyrus, with lesion severity rated as ++ in two cases and + in one case. In the IR + memantine group, the lesion severity was ++ in one case and + in two cases, indicating a modest reduction in damage compared to the IR group. The IR + HRW group showed a similar trend, with severity rated as ++ in one case and + in one case, while the IR + joint group exhibited the mildest changes among the irradiated groups, with ++ in only one case. Compared to the control group, which displayed no significant abnormalities, the IR group demonstrated the most severe histopathological lesions. The IR + memantine group exhibited slightly reduced lesion severity, while the IR + HRW and IR + joint groups demonstrated further improvements. Notably, the IR + joint group showed the least severe lesions among the irradiated groups. The overall severity ranking of hippocampal lesions was as follows: IR group > IR + memantine group > IR + HRW group > IR + joint group > control group (Figure 3a).

#### 3.3.2. BrdU + NeuN Immunofluorescence Double Labeling

BrdU + NeuN immunofluorescence double labeling was used to assess neuronal maturity and neurogenesis. NeuN, which labels mature neurons, revealed sparse, loose, and disorganized neurons in the IR group, whereas the control group exhibited dense and orderly neuronal organization. All treatment groups demonstrated varying degrees of improvement in neuronal arrangement compared to the IR group. BrdU, which marks newly generated neurons, showed minimal compensatory neurogenesis in the control group, likely due to functional integrity and a higher proportion of mature neurons. Among the irradiated groups, the number of newly generated neurons ranked from highest to lowest as follows: IR + Joint > IR + memantine > IR + HRW > IR (Figure 3b).

#### 3.3.3. Transmission Electron Microscopy (TEM) Analysis

Transmission electron microscopy (TEM) revealed significant ultrastructural changes in irradiated cells. In the IR group, cells exhibited severe damage, including localized disruption of the cell membrane, sparse and dissolved intracellular matrix, reduced electron density, organelle swelling, and vacuolization. The IR + memantine and IR + HRW groups displayed less severe damage. Specifically, the IR + memantine group exhibited pronounced edema with intact cell membranes, a uniform intracellular matrix, and noticeable organelle swelling, clustering, and extensive vacuolization. The IR + HRW group predominantly showed cytoplasmic edema, localized membrane disruption and dissolution (indicated by arrows), a sparse and dissolved intracellular matrix, and large areas of low electron density. In contrast, the IR + Joint group demonstrated moderate cellular damage, with intact and continuous cell membranes, a uniform intracellular matrix, a moderate number of organelles, and moderate swelling. The severity of cellular damage, ranked from most to least, was as follows: IR > IR + memantine ≥ IR + HRW > IR + Joint (Figure 3c).

#### 3.3.4. Golgi Staining

**Figure 4:**
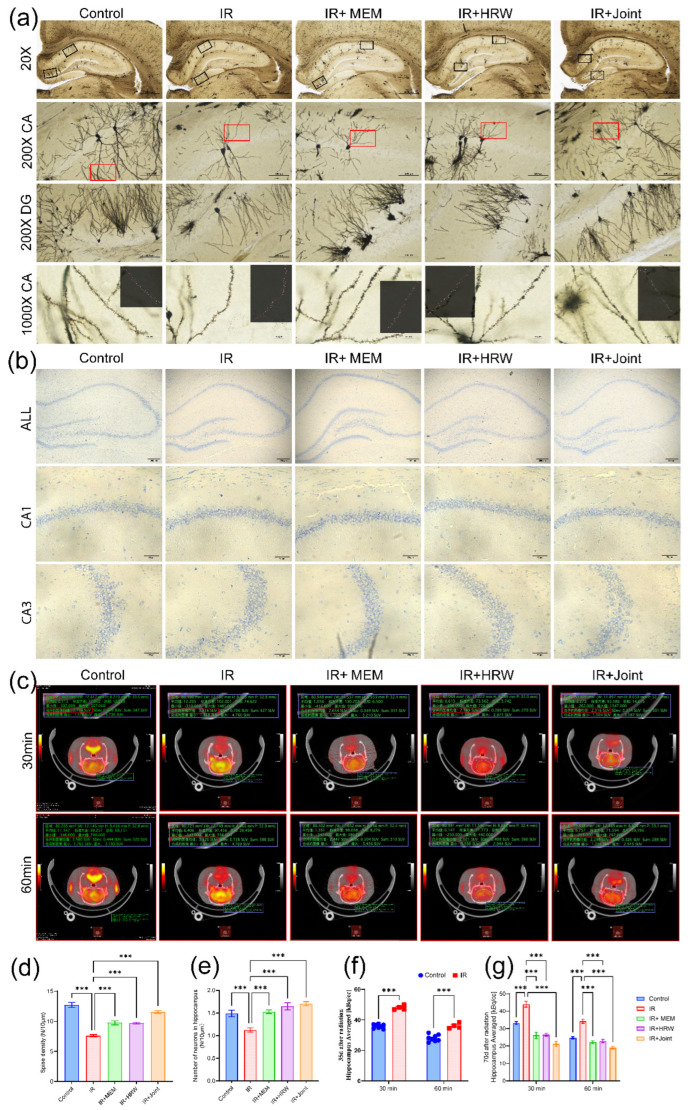
Effects of chronic irradiation on hippocampal neuronal morphology and glucose metabolism. (**a**) Golgi staining of rat hippocampus, showing depth-of-field images (*n* = 3). (**b**) Nissl staining of rat hippocampus under light microscopy. (**c**) Cross-sectional ^18^F-FDG PET/CT images of the hippocampus on day 70 post-irradiation. (**d**) Dendritic spine density in the CA region of rat hippocampus after Golgi staining (*n* = 3). (**e**) Neuronal count per unit length in the CA1 region of rat hippocampus after Nissl staining (*n* = 3 × 2 sides). (**f**) ^18^F-FDG uptake activity concentration in the hippocampal region at day 35 post-irradiation. (**g**) ^18^F-FDG uptake activity concentration in the hippocampal region at day 70 post-irradiation. (*** *p* < 0.001).

Golgi staining revealed significant neuronal changes in the IR group, including neuronal reduction, atrophy, decreased branching, and a notable reduction in dendritic and dendritic spine density, as illustrated in Figure 4a. Binary segmentation analysis was performed on dendrites, and dendritic spines on branches of the same order were quantified to calculate the spine density per micrometer. Compared to the IR group, all treatment groups (IR + memantine, IR + HRW, and IR + Joint) demonstrated significant improvements in dendritic and dendritic spine density (*p* < 0.001, Figure 4d).

#### 3.3.5. Nissl Staining

The results of Nissl staining revealed a significant reduction in neuronal numbers in the IR group, with neurons exhibiting a loose arrangement. In contrast, the intervention groups (IR + memantine, IR + HRW, and IR + Joint) showed marked improvements, as shown in Figure 4b. Quantitative analysis of neurons in the CA1 region demonstrated that the control group, IR + memantine group, IR + HRW group, and IR + joint group had significantly higher neuronal counts per unit length compared to the IR group, with statistically significant differences (*p* < 0.001). Notably, the IR + Joint group showed the most significant improvement, as shown in Figure 4e.

### 3.4. ^18^F-FDG PET/CT Brain Static Imaging and Data Reconstruction Analysis

The ^18^F-FDG PET/CT brain static imaging results revealed that on day 35 post-irradiation, glucose uptake in the hippocampal region was significantly higher in the IR group compared to the control group at both 30 and 60 min after FDG injection (*p* < 0.001, Figure 4f). Based on these observations, all groups underwent ^18^F-FDG PET/CT imaging again on day 70 post-irradiation. Cross-sectional images demonstrated that the brain’s standard uptake value (SUV) in the IR group remained higher than in the other groups, Figure 4c. Co-registration of PET/CT with MRI further confirmed that the ^18^F-FDG activity concentration in the hippocampal region was significantly lower in the control and intervention groups compared to the IR group (*p* < 0.001), as shown in Figure 4g. These differences may be attributed to chronic brain inflammation and blood–brain barrier (BBB) damage induced by irradiation.

### 3.5. Oxidative Stress Markers

Oxidative stress markers, including GSH, ^•^OH, and SOD, were measured in brain tissue. The results showed minimal variation among the groups overall. However, the IR + memantine group exhibited a statistically significant increase in GSH levels compared to the IR group (*p* < 0.05), while the IR + Joint group demonstrated a significantly higher SOD level than the IR group (*p* < 0.05). No statistically significant differences were observed in the other groups (*p* > 0.05), as shown in Figure S3. These results suggest that during the chronic phase of radiation-induced brain injury (RIBI), oxidative stress markers may have partially recovered, leading to less pronounced changes compared to those typically observed in the acute phase.

### 3.6. Transcriptomic Findings in the Hippocampus

Based on previous experimental results, the IR + HRW group demonstrated a certain degree of efficacy in alleviating chronic radiation-induced brain injury (RIBI) compared to the IR group. To further explore the potential molecular mechanisms underlying HRW’s effects, transcriptomic sequencing was performed on hippocampal tissue at the molecular level.

#### 3.6.1. Screening of Differentially Expressed Genes (DEGs)

**Figure 5:**
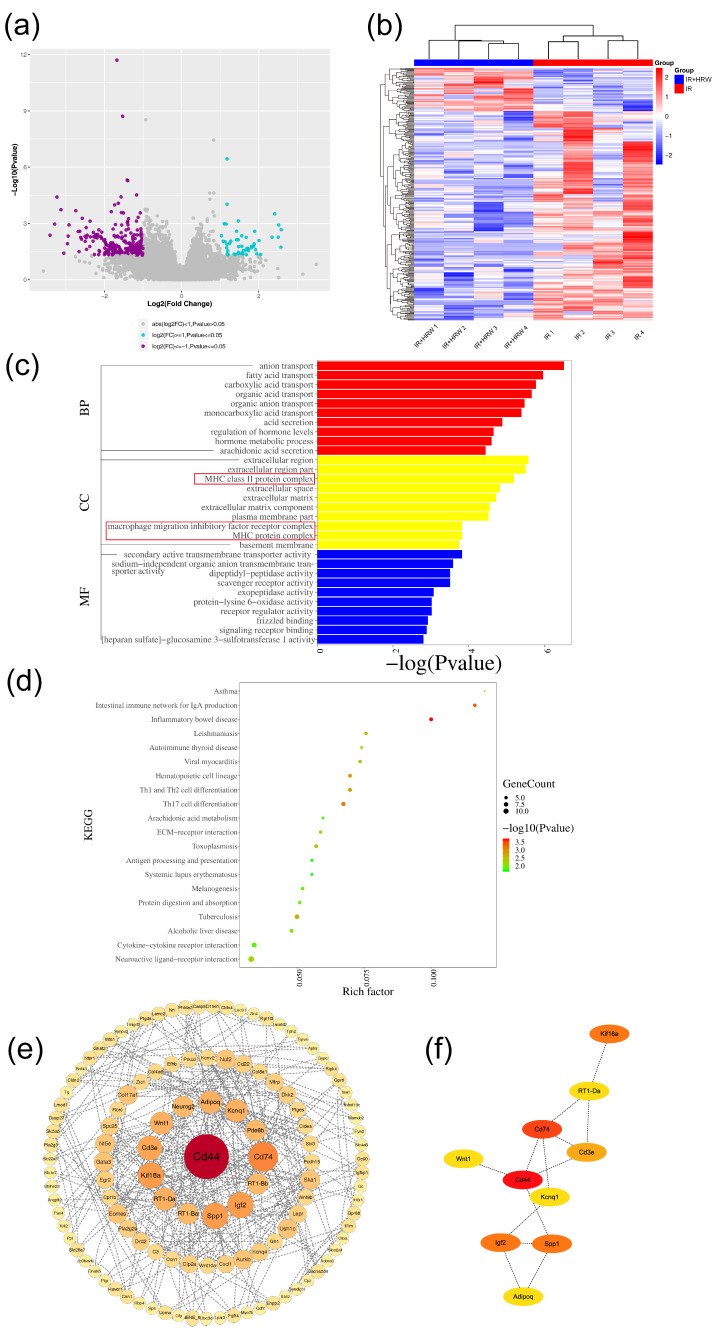
Transcriptomic analysis of the IR + HRW group compared to the IR group. (**a**) Volcano plot showing differentially expressed genes (DEGs) between the groups. (**b**) Heatmap of DEG clustering. (**c**) Bar plot of top 10 significantly enriched Gene Ontology (GO) terms (*p* ≤ 0.05). (**d**) Bubble plot of top 20 enriched GO terms, with the rich factor on the x-axis. (**e**) Protein–protein interaction (PPI) network, with node size and color reflecting interaction degree. (**f**) Sub-network of the top 10 hub genes in the PPI network, with node color indicating connectivity level.

A volcano plot and scatter plot were used to identify significant differentially expressed genes (DEGs) between the IR and IR + HRW groups, where genes with ∣log2FC∣ ≥ 1 and *p* ≤ 0.05 were considered significant. Upregulated genes (log2FC ≥ 1) were shown as blue dots, downregulated genes (log2FC ≤ −1) as purple or red dots, and non-significant genes in gray. The analysis identified 298 DEGs, including 247 downregulated and 51 upregulated genes in the IR + HRW group compared to the IR group (Figure 5a). Notably, genes associated with pyroptosis pathways, previously identified in the acute phase, were absent, suggesting that chronic radiation-induced brain injury may not involve pyroptosis pathways or that pyroptosis had resolved during the chronic phase. A hierarchical clustering heatmap further demonstrated that most DEGs in the IR group exhibited higher expression levels compared to the IR + HRW group, as indicated by red (high expression) and blue (low expression) color gradients (Figure 5b).

#### 3.6.2. GO Enrichment Analysis of Differentially Expressed Genes

To explore the functional roles of the identified DEGs, Gene Ontology (GO) enrichment analysis was performed, categorizing DEGs into three domains: biological process (BP), cellular component (CC), and molecular function (MF). A total of 1053 GO terms were enriched, with 843 BP terms (80.0%), 61 CC terms (5.7%), and 149 MF terms (14.1%). Detailed results, including specific pathways and statistical significance, are presented in Figure 5c.

#### 3.6.3. KEGG Enrichment Analysis of Differentially Expressed Genes

KEGG enrichment analysis was performed on the 298 differentially expressed genes (DEGs) using the clusterProfiler package in R. Similar to GO analysis, Fisher’s exact test was applied to determine whether KEGG pathways were significantly enriched, with a significance threshold of *p* ≤ 0.05. A total of 36 pathways were identified, and the top 20 pathways, ranked by *p*-value, are shown in Figure 5d. These pathways are predominantly related to inflammation and immune response, including Inflammatory Bowel Disease, Intestinal Immune Network for IgA Production, Th17 Cell Differentiation, Hematopoietic Cell Lineage, and Th1 and Th2 Cell Differentiation.

#### 3.6.4. Gene Set Enrichment Analysis (GSEA)

In the hippocampal tissue of rats from the IR + HRW group compared to the IR group, Gene Set Enrichment Analysis (GSEA) revealed significant molecular regulatory mechanisms. Within the C2 (curated gene sets) and C5 (GO gene sets) collections, pathways significantly upregulated in the IR + HRW group included Oxidative Phosphorylation, Respiratory Electron Transport, Mitochondrial Translation, KEAP1-NFE2L2 Pathway, Formation of TC-NER Pre-Incision Complex, G1/S DNA Damage Checkpoints, Stabilization of P53, and Translation. These upregulated pathways indicate that the drug mitigates radiation damage by enhancing energy metabolism, antioxidant defenses, DNA repair, and protein synthesis, thereby supporting the recovery of hippocampal neurons. Conversely, pathways such as Extracellular Matrix Organization, Degradation of the Extracellular Matrix, Collagen Formation, TGF-β Signaling Pathway, Asthma, and Response to radiation were significantly downregulated, suggesting that the drug suppresses excessive ECM remodeling, fibrosis, and inflammation, protecting hippocampal structural integrity and reducing neuroinflammatory damage (Figure S4a–d). In summary, HRW significantly alleviates radiation-induced brain injury by upregulating protective pathways to enhance cellular resilience while downregulating pathways associated with pathological remodeling and inflammation, offering potential therapeutic targets for neuroprotection.

#### 3.6.5. Protein–Protein Interaction (PPI) Network Analysis

The 298 differentially expressed genes (DEGs) were uploaded to the STRING database (https://cn.string-db.org/, accessed on 11 December 2023) for PPI network analysis,. After removing isolated nodes, the PPI data were analyzed in Cytoscape software to identify key genes based on degree, defined as the number of edges connected to each node (Figure 5e). The subnetwork of the top 10 hub genes was constructed and is displayed in Figure 5f. Based on the PPI network results, the following genes were selected for further validation: Cd44, Cd74, Cd3e, RT1-Ba, RT1-Da, Spp1, Adipoq, Kif18a, and Wnt1.

### 3.7. Real-Time Quantitative PCR Validation of Key Protein-Related Gene Expression

**Figure 6:**
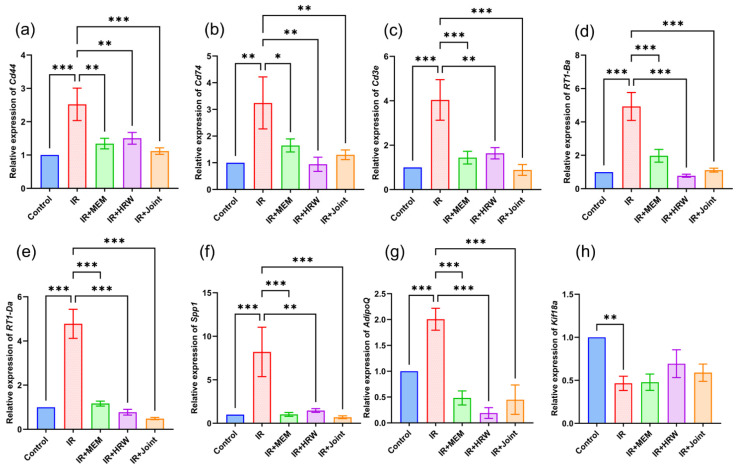
Real-time quantitative PCR validation of key protein-related gene mRNA expression in different groups (*n* = 5). (**a**) Relative expression level of *Cd44*, (**b**) relative expression level of *Cd74*, (**c**) relative expression level of *Cd3e*, (**d**) relative expression level of *RT1-Ba*, (**e**) relative expression level of *RT1-Da*, (**f**) relative expression level of *Spp1*, (**g**) relative expression level of *Adipoq*, (**h**) relative expression level of *Kif18a.* Statistical significance is indicated as * *p* < 0.05, ** *p* < 0.01, *** *p* < 0.001 compared with the IR group.

The RT-qPCR results for hippocampal tissue at 74 days post-irradiation are shown in Figure 6. Compared to the control group, the IR group exhibited significantly higher mRNA expression levels of CD44, CD74, CD3e, RT1-Ba, RT1-Da, SPP1, and AdipoQ, while Kif18A showed significantly lower expression (*p* < 0.05). Compared to the IR group, the expression levels of CD44, CD74, CD3e, RT1-Ba, RT1-Da, SPP1, and AdipoQ were significantly reduced in the IR + memantine, IR + HRW, and IR + joint groups (*p* < 0.05).

### 3.8. Western Blot Analysis

**Figure 7:**
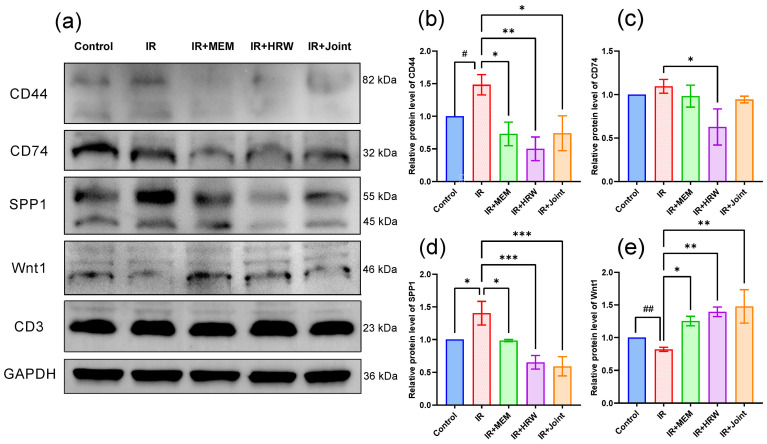
Western blot analysis of key proteins in the rat model of chronic radiation-induced brain injury (RIBI). (**a**) Western blotting results showing the expression of CD44, CD74, SPP1, and Wnt1 proteins in rat brain tissue. (**b**–**e**) Quantitative analysis of the relative expression levels of CD44 (**b**), CD74 (**c**), SPP1 (**d**), and Wnt1 (**e**) normalized to GAPDH. Data are presented as mean ± SEM (*n* = 3). # *p* < 0.05, ## *p* < 0.01: unpaired two-tailed Student’s t-test between control and IR groups. * *p* < 0.05, ** *p* < 0.01, *** *p* < 0.001: one-way ANOVA followed by uncorrected LSD post hoc tests vs. IR group.

Western blot analysis of hippocampal tissue following ionizing radiation revealed significant changes in protein expression levels among the groups. Compared to the IR group, the protein levels of CD44, and SPP1 were significantly reduced in the intervention groups (Figure 7a,b,d, *p* < 0.05). Specifically, CD44 expression was significantly lower in the IR + HRW and IR + joint groups, while CD74 expression was significantly reduced in the IR + HRW group (Figure 7c, *p* < 0.05). SPP1 levels were significantly lower in all intervention groups compared to the IR group (*p* < 0.05). In contrast, the expression of Wnt1 was significantly higher in the IR + HRW and IR + joint groups compared to the IR group (Figure 7e, *p* < 0.05). No significant differences were observed in CD3 protein levels across the groups (Figure 7).

### 3.9. Serum ELISA Biomarker Detection

**Figure 8:**
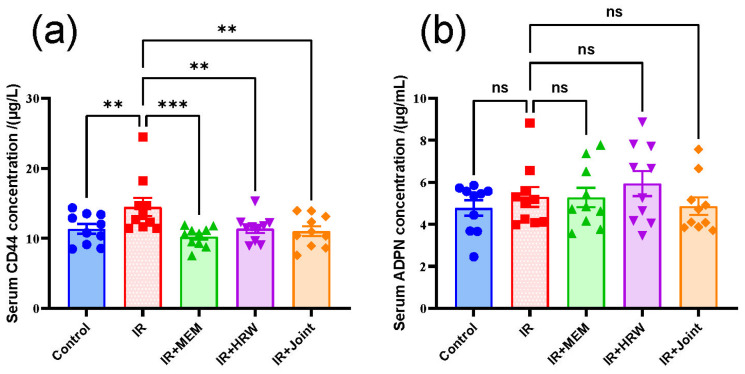
Serum levels of CD44 and ADPN in rats. (**a**) CD44 and (**b**) ADPN protein concentrations in rat serum (*n* = 10 per group). Data are presented as mean ± SEM. Statistical significance is indicated as ns: not significant, ** *p* < 0.01, *** *p* < 0.001 compared with the IR group.

To investigate whether the key proteins CD44 and ADPN (encoded by the *Adipoq* gene) in rat serum could serve as potential biomarkers for chronic radiation-induced brain injury (RIBI), ELISA was performed following transcriptomic screening, RT-qPCR, and Western blot analysis. The results showed that serum CD44 levels were significantly lower in the control and intervention groups compared to the IR group (Figure 8a, *p* < 0.01). However, no significant differences were observed in serum ADPN levels among the groups (Figure 8b, *p* > 0.05).

## 4. Discussion

In our previous study, we demonstrated that memantine and hydrogen-rich water (HRW) exert protective effects during acute radiation-induced brain injury (RIBI) by regulating the NLRP3/NLRC4/Caspase-1 pyroptosis pathway, alleviating neuroinflammation and brain injury [20]. However, the pathological mechanisms and therapeutic strategies for chronic RIBI remain largely unclear due to its complexity. This study further explores the long-term protective effects and potential molecular mechanisms of HRW in chronic RIBI, providing a theoretical foundation for clinical translation. Chronic radiation-induced brain injury is clinically classified as a late-delayed response, typically manifesting six months or more after radiotherapy. It is the most common form of RIBI, often referred to as late radiation brain injury, and predominantly occurs in patients receiving radiation doses exceeding 50 Gy to the brain. The condition is characterized by focal brain symptoms, cortical dysfunction, intracranial hypertension, and hypothalamic–pituitary axis abnormalities, with clinical manifestations such as headaches, cognitive dysfunction, seizures, and neurological deficits (e.g., limb numbness), which represent newly developed symptoms of brain injury after radiotherapy [25,26]. Based on time equivalence between humans and rodents, chronic RIBI in rats is typically defined as brain injury occurring 30 days post-irradiation. Using this animal model, the present study further investigates the long-term protective effects of HRW and its underlying molecular mechanisms in mitigating chronic RIBI.

### 4.1. Behavioral Improvements by HRW in Chronic RIBI

Radiation-induced brain injury (RIBI) severely affects cognitive function and quality of life. Behavioral tests were used to evaluate the effects of hydrogen-rich water (HRW) intervention in mitigating cognitive deficits caused by chronic RIBI. In the open field test, the IR group showed significantly reduced central area activity at 30 and 60 days post-irradiation, indicating anxiety-like behavior. In contrast, the control and IR + HRW groups exhibited significantly increased central activity, suggesting that HRW alleviates radiation-induced anxiety-like behavior. In the Y-maze test, the IR group had fewer spontaneous alternations and lower exploration scores compared to the control and IR + HRW groups. These results indicate that HRW improves spatial working memory and exploratory behavior impaired by irradiation. In the Morris Water Maze (MWM), HRW intervention significantly improved spatial learning and memory. Compared to the IR group, the IR + HRW group showed shorter escape latencies during the navigation trial and better performance in the probe tests on days 6 and 8, demonstrating HRW’s protective effect on spatial memory and orientation.

### 4.2. Histopathological Analysis of HRW’s Protective Effects

Histopathological evaluation revealed that hydrogen-rich water (HRW) mitigated radiation-induced brain injury (RIBI) by alleviating structural damage in the hippocampus. HE staining showed that the IR group had disorganized neuronal arrangements and swelling degeneration in the dentate gyrus, which were significantly improved in the HRW group. The severity of damage followed the order IR > IR + memantine > IR + HRW > IR + joint > control. Nissl staining demonstrated a reduction in neuronal count and loose neuronal arrangements in the IR group, while the HRW group exhibited significantly higher neuronal counts and improved structural organization. Golgi staining revealed a marked decrease in dendritic spine density in the IR group, which was significantly restored in the HRW group. Additionally, BrdU + NeuN double immunofluorescence staining showed that the number of newly generated and mature neurons was significantly higher in the HRW group compared to the IR group. At the ultrastructural level, transmission electron microscopy (TEM) revealed subcellular damage in the IR group, including disrupted membranes, sparse intracellular matrix, reduced electron density, and organelle swelling. These pathological changes were notably alleviated by HRW intervention. Collectively, these findings suggest that RIBI and its associated cognitive impairments may be linked to neuronal swelling, reduced neuronal count and spine density, decreased neurogenesis, and subcellular damage, all of which were mitigated by HRW treatment.

### 4.3. Regulation of Oxidative Stress and Inflammation by HRW

Oxidative stress indicators measured at 74 days post-irradiation showed that changes in MDA, SOD, and OH^•^ levels during the chronic phase were far less pronounced compared to the acute phase, likely due to compensatory recovery mechanisms. ^18^F-FDG PET/CT static brain imaging and data reconstruction analysis revealed that hippocampal FDG uptake was significantly higher in the IR group compared to other groups. This difference may be attributed to chronic brain inflammation and blood–brain barrier (BBB) damage. It is well established that false positives in PET imaging are often associated with inflammatory responses, as inflamed regions exhibit increased glucose uptake (elevated F-18 activity). Previous studies have also reported that RIBI can lead to BBB disruption, which might further contribute to increased glucose uptake. Clinically, PET imaging is commonly used to differentiate radiation-induced brain injury from tumor recurrence, with a sensitivity of 80–90% and a specificity of 50–90% [27].

### 4.4. Transcriptomic Analysis Reveals Potential Molecular Mechanisms

Transcriptomic sequencing identified 298 differentially expressed genes (DEGs) between the IR and IR + HRW groups, including 247 downregulated and 51 upregulated genes in the IR + HRW group (|log2FC| ≥ 1, *p* ≤ 0.05). Gene Set Enrichment Analysis (GSEA) indicated that HRW treatment significantly upregulated pathways involved in oxidative phosphorylation, mitochondrial translation, DNA repair, and antioxidant defense while downregulating pathways related to extracellular matrix remodeling, TGF-β signaling, and inflammation. These findings suggest that HRW may alleviate radiation-induced damage by enhancing cellular resilience and suppressing pathological responses.

Subsequent protein–protein interaction (PPI) network analysis identified key hub genes such as *CD44*, *CD74*, *CD3e*, *RT1-Ba*, *RT1-Da*, *SPP1*, *AdipoQ*, *KIF18A*, and *Wnt1* for further validation. Notably, genes associated with pyroptosis, which were prominent during the acute phase, were not differentially expressed in either control vs. IR or IR vs. IR + HRW comparisons during the chronic stage. This suggests that pyroptosis may not play a major role in the chronic phase of radiation-induced brain injury.

### 4.5. Pathway Analysis: Exploring the Mechanisms of HRW

RT-qPCR and Western blotting were used to validate key genes and proteins identified in the transcriptomic analysis, showing results consistent with the transcriptomic data. After ionizing radiation, hippocampal tissues exhibited elevated expression levels of CD44, CD74, CD3e, RT1-Ba, RT1-Da, SPP1, and AdipoQ, while HRW intervention significantly reduced these levels compared to the IR group.

CD44 is a complex molecular aggregate associated with various inflammatory and tumor-related diseases. CD74, a protein linked to the class II major histocompatibility complex (MHC), is an essential chaperone protein for antigen presentation and also functions as a cell surface receptor for the cytokine macrophage migration inhibitory factor (MIF).

In rats, the major histocompatibility complex (MHC) is referred to as the RT1 complex. Similar to the MHC of other species, the RT1 complex is composed of a tightly linked cluster of genes, including class I, class II, and class III genes [28]. Among these, RT1-B/D constitutes the class II MHC (MHC II). In our RT-qPCR results, the expression trends of RT1-Ba and RT1-Da genes were consistent across groups, suggesting that these two genes may share similar expression levels.

The MIF signaling pathway was also identified in our GO enrichment analysis. MIF is a pro-inflammatory cytokine encoded by a polymorphic functional genetic locus. It not only exerts pro-inflammatory effects as a cytokine but also functions as a chemokine, regulating the production of other pro-inflammatory cytokines [29]. As a key upstream mediator of innate and adaptive immune responses and survival pathways, MIF plays a protective role in clearing pathogens during infections. However, as an immunomodulator, MIF also exacerbates harmful inflammation, promotes cancer metastasis and progression, and worsens disease conditions [29]. MIF exerts its diverse biological functions primarily through interactions with membrane receptors of the CXC family, CD74, and CD44, thereby activating downstream signaling pathways [30]. Pantouris et al. [31] demonstrated that CD74, a membrane protein, is a high-affinity receptor for MIF. MIF can regulate CD74 activity, leading to homeostatic imbalances such as inflammation, tumors, and autoimmune diseases. By interacting with CD74, MIF activates the MAPK signaling pathway and persistently activates ERK signaling. Rajasekaran et al. [32] reported that MIF binds to chemokine receptors CXCR2 and CXCR4, recruiting leukocytes and accelerating the progression of atherosclerosis.

Further studies reveal that these membrane receptors often form complexes (e.g., CXCR4/CD74, CXCR2/CD74, CD74/CD44) with MIF to regulate cellular functions. CD74, a transmembrane glycoprotein involved in MHC II antigen presentation, primarily serves to stabilize antigen peptides. However, it lacks an intracellular active domain and requires interaction with CD44 to complete signal transduction. CD44 itself lacks phosphatase activity. When MIF binds to the CD74/CD44 complex, serine phosphorylation is induced, which activates the Src tyrosine kinase pathway, thereby mediating signal transduction. Additionally, the MIF-CD74/CD44 complex also activates Syk, PI3K, Akt, ERK1/2, and NF-κB signaling pathways, thereby regulating immune responses [33]. Most of these pathways are involved in stress responses, inflammation, and cell death. Importantly, Wang et al. demonstrated that CD44 deficiency markedly reduces proinflammatory cytokine expression in microglia and astrocytes, ameliorates motor deficits, and preserves dopaminergic neurons in a Parkinson’s disease mouse model. These protective effects are likely mediated by suppression of the TLR4/NF-κB signaling axis [34], underscoring CD44′s key regulatory role in neuroinflammation and its potential as a therapeutic target.

The SPP1 protein, officially named secreted phosphoprotein-1 (SPP1) and also known as osteopontin (OPN), is a multifunctional glycoprotein initially identified as a pro-inflammatory cytokine secreted by T cells. Subsequent studies revealed that resident macrophages in various tissues also express SPP1, which is closely involved in processes such as apoptotic cell clearance, chemotaxis, and macrophage migration [35]. CD44 is one of the receptors for osteopontin SPP1 [36]. Cheng et al. [37] discovered that the SPP1-CD44 interaction mediates communication with T cells, inhibiting their proliferation and contributing to tumor immune escape, ultimately resulting in poorer patient survival rates. He et al. [38] reported that targeting the SPP1-CD44 axis restored T-cell functionality in vitro. In mouse models, the use of anti-SPP1 or anti-CD44 antibodies, either alone or in combination with PD-1 antibodies, significantly reduced tumor burden. A report in *Nature Neuroscience* showed that SPP1 promotes synaptic phagocytosis by microglia in Alzheimer’s disease (AD). In hippocampal tissue from AD patients, SPP1 expression is significantly increased, while SPP1 knockout reduces microglial phagocytic activity and inhibits synaptic loss [35]. Microglia are a type of resident mononuclear macrophage in the central nervous system (CNS) [39]. Moreover, Chow et al. [40] reported that macrophages suppress the antitumor function of CD8+ T cells. Collectively, inhibiting SPP1 expression may attenuate macrophage phagocytosis and restore T-cell function. Furthermore, Al-Dalahmah et al. [41] revealed that osteopontin drives neuroinflammation and neuronal loss in frontotemporal dementia patients. Our results also demonstrated that ionizing radiation significantly increased SPP1 expression in hippocampal tissue, while SPP1 expression in groups treated with hydrogen-rich water (HRW) and memantine was significantly lower than in the IR group. These findings suggest that HRW intervention may suppress inflammation and synaptic loss, thereby improving cognitive function. Whether HRW can restore T-cell function and improve tumor immune escape remains to be further investigated.

CD3e is a single-pass type I membrane glycoprotein expressed on T-cell surfaces. As a key leukocyte differentiation antigen, CD3 is present on nearly all T cells, and CD3e plays an important role in adaptive immune responses. Deng et al. [42] demonstrated in cells that the CD3e ligand (CD3L1) inhibits T-cell activation. In our RT-qPCR experiments, CD3e expression was elevated in the IR group but low in the control and IR + HRW groups.

Adiponectin (ADPN), encoded by the *AdipoQ* gene, is present in cerebrospinal fluid (CSF) at concentrations approximately 0.1% of those in serum [43]. When the blood–brain barrier (BBB) is damaged, ADPN levels in the brain and CSF increase, suggesting its critical role in the CNS. Une et al. [44] found elevated plasma and CSF adiponectin levels in patients with mild cognitive impairment and Alzheimer’s disease (AD), indicating its involvement in AD pathogenesis. Moreover, a cohort study revealed that blood adiponectin levels positively correlated with increased brain amyloid deposition, thereby promoting AD progression [45]. Conversely, some studies suggest that ADPN reduces inflammatory markers such as C-reactive protein (CRP), interleukin-6 (IL-6), and tumor necrosis factor-α (TNF-α), exerting neuroprotective effects [46]. Zhang et al. reported that ADPN not only prevents brain atrophy and neurofunctional decline but also promotes angiogenesis after stroke [47]. This dual role of adiponectin in neuronal protection and disease promotion is termed the “adiponectin paradox” [48]. Many researchers consider it a “rescue hormone” [49], whose elevated levels represent a compensatory response to combat disease. Our results showed significantly increased ADPN expression in hippocampal tissue of the IR group, whereas low expression was observed in the control and IR + HRW groups, suggesting its detrimental role in IR-induced cognitive impairment. In serum, no significant differences in ADPN levels were detected among groups. Combined with PET/CT findings, we hypothesize that ionizing radiation damages the BBB, allowing ADPN to cross from plasma into the brain. HRW may alleviate BBB damage and mitigate this adverse effect.

Wnt, a secreted glycoprotein, exerts its effects by binding to specific seven-transmembrane receptors of the Frizzled (Fz) family, thereby activating multiple signaling pathways [50,51]. The Wnt pathway is critical for embryonic development, neurogenesis, synapse formation, and neuronal plasticity, playing essential roles in advanced brain functions such as learning and memory [50]. Several studies have demonstrated that Wnt1 signaling improves learning and memory deficits [52,53]. In our study, Wnt1 protein expression was significantly upregulated in the HRW and combined treatment groups compared to the IR group. Behavioral indicators in the HRW group were also markedly better than in the IR group, suggesting that HRW may improve radiation-induced brain injury and cognitive deficits via the Wnt1 pathway. Consistent with our findings, previous studies have shown that upregulation of Wnt/β-catenin signaling can exert protective effects in disease models such as diabetic nephropathy, where pharmacological activation of suppressed Wnt proteins—such as Wnt1, Wnt4, and Wnt5a—was associated with reduced cellular damage and fibrosis [54].

### 4.6. Preliminary Exploration of Early Biomarkers for Chronic RIBI

In our experiments, serum CD44 levels were lower in the Control group and all intervention groups compared to the IR group. This aligns with a report in *Nature Medicine* suggesting that CD44 could serve as a cerebrospinal fluid (CSF) biomarker for Alzheimer’s disease (AD) [55]. However, the conditions for defining biomarkers are relatively stringent. Whether CD44 can serve as a biomarker for chronic radiation-induced brain injury (chronic RIBI) remains to be further investigated.

### 4.7. Study Limitations and Translational Considerations

While this study provides compelling evidence for the neuroprotective effects of hydrogen-rich water (HRW) in a rat model of chronic RIBI, there are inherent limitations in translating these findings directly to humans. Differences in brain anatomy, radiation sensitivity, pharmacokinetics, and immune response between rodents and humans must be considered. Furthermore, the efficacy, optimal dosage, and long-term safety profile of HRW in human patients remain to be validated through well-designed clinical trials. Therefore, while our results offer important mechanistic insights and therapeutic potential, further research is necessary before HRW can be confidently applied in clinical practice.

## 5. Conclusions

This study demonstrates that hydrogen-rich water (HRW) significantly alleviates chronic radiation-induced brain injury (RIBI) and associated cognitive deficits in rats. These protective effects are linked to improvements in neuronal morphology, enhanced neurogenesis, restoration of dendritic spine density, and reduced subcellular damage. Transcriptomic and molecular analyses reveal that HRW modulates key genes such as *CD44*, *CD74*, *SPP1*, and *Wnt1* and exerts its effects through pathways including MIF, Wnt, and SPP1 signaling. These mechanisms collectively contribute to reduced neuroinflammation, improved synaptic integrity, and enhanced neuroplasticity. Importantly, our findings suggest that HRW, a non-toxic and accessible intervention, holds promise as a therapeutic strategy for mitigating RIBI. Moreover, serum CD44 may serve as a potential biomarker for chronic RIBI, warranting further investigation. This study provides novel insights into the molecular mechanisms of chronic RIBI and lays a foundation for translational research on HRW-based interventions.

## Figures and Tables

**Table 1:** Rat entry positions during the Morris Water Maze experiment.

Day	Trial 1	Trial 2	Trial 3	Trial 4
1	S	W	NW	SE
2	NW	S	SE	W
3	SE	NW	W	S
4	W	SE	S	NW
5	S	NW	W	SE
6 & 8 (Probe)	SW			

**Table 2:** Primer sequences.

Gene Name	Accession Number	Primer (5′ to 3′)	Product Length
*Cd44*	NM_012924.3	Forward primer: TCGATTTGAATATAACCTGCCG Reverse primer: CAGTCCTGGAGATACTGTAGC	78
*Cd74*	XM_006254761.4	Forward primer: CACCCAGACTCCACCTAAAGTATT Reverse primer: TCTCATCACACTTGGGACGG	98
*Cd3e*	NM_001108140.2	Forward primer: CCAGACTATGAGCCCATCCG Reverse primer: TAGGATGCGTGTTCACCAGG	178
*RT1-Ba*	NM_001008831.3	Forward primer: TCCCCGAGTTTGGACAACTG Reverse primer: GTCGCCTCAGGAACCTTGTT	133
*RT1-Da*	NM_001008847.2	Forward primer: CTGTGAGATACCAGGAGGTGATG Reverse primer: AGGGGTATCCTCAGATGCTGT	79
*Spp1*	NM_012881.2	Forward primer: GCCAGCCAAGGACCAACTAC Reverse primer: AGTGTTTGCTGTAATGCGCC	133
*Adipoq*	NM_144744.3	Forward primer: AATCCTGCCCAGTCATGAAG Reverse primer: TCTCCAGGAGTGCCATCTCT	159
*Kif18a*	NM_001137642.1	Forward primer: TGCCTTAGCAGATACAAAGAGAAGA Reverse primer: GTCTTTGGCACGATTTGCGT	184
*Gapdh*	NM_017008.4	Forward primer: CCGCATCTTCTTGTGCAGTG Reverse primer: CGATACGGCCAAATCCGTTC	79

## Data Availability

The data presented in this study are available on reasonable request from the corresponding author.

## Supplementary Materials

The following supporting information can be downloaded at https://www.mdpi.com/article/10.3390/antiox14080948/s1. Figure S1: Brain localization and irradiation setup. Figure S2: Body weight and food intake. Figure S3: Brain tissue GSH, ^•^OH, and SOD levels. Figure S4: Gene Set Enrichment Analysis (GSEA).
