# Supplementary material for: Long-Term Neuroprotective Effects of Hydrogen-Rich Water and Memantine in Chronic Radiation-Induced Brain Injury: Behavioral, Histological, and Molecular Insights
Source: Antioxidants (Basel). 2025 Aug 1;14(8):948. doi: 10.3390/antiox14080948 (PMC12382694; doi:10.3390/antiox14080948)
Supplement: Supplementary file 1 [file antioxidants-14-00948-s001.zip › antioxidants-3778662-supplementary.pdf]

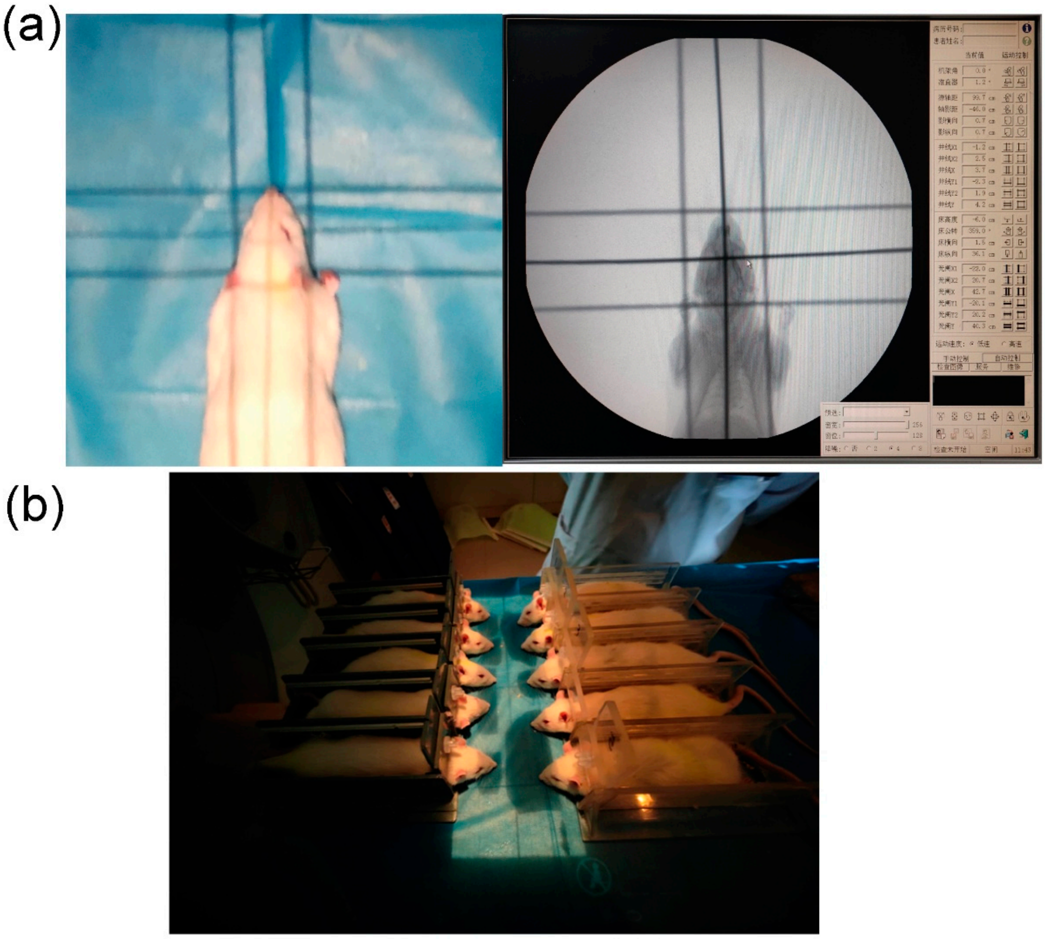

Figure S1. Brain localization and irradiation setup. (a) Schematic of intracranial brain localization in rats. (b) Irradiation setup for the experiment.

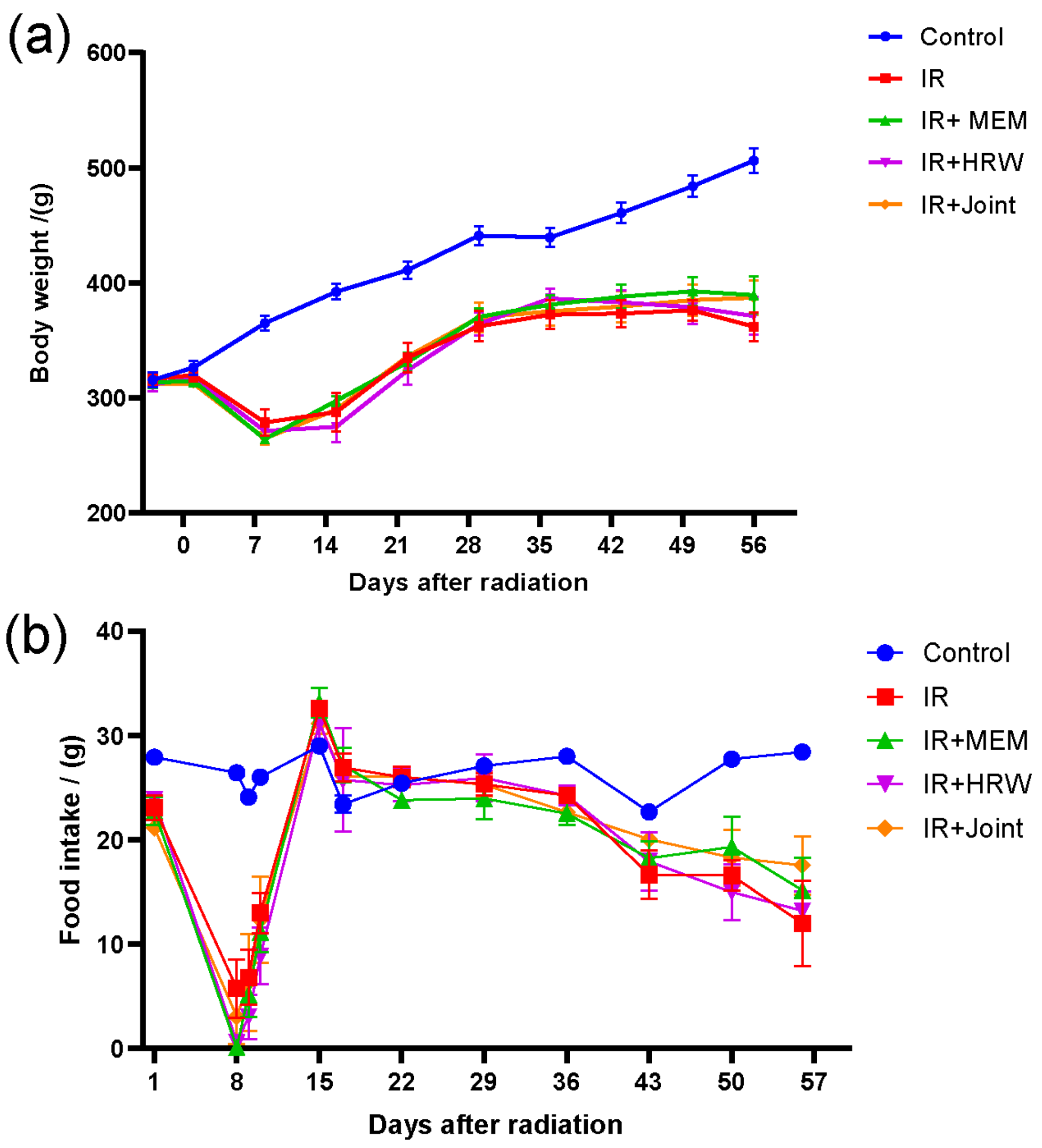

Figure S2. Body weight and food intake. (a)Results of Body weight Measurements. (b)Results of food intake Measurements.

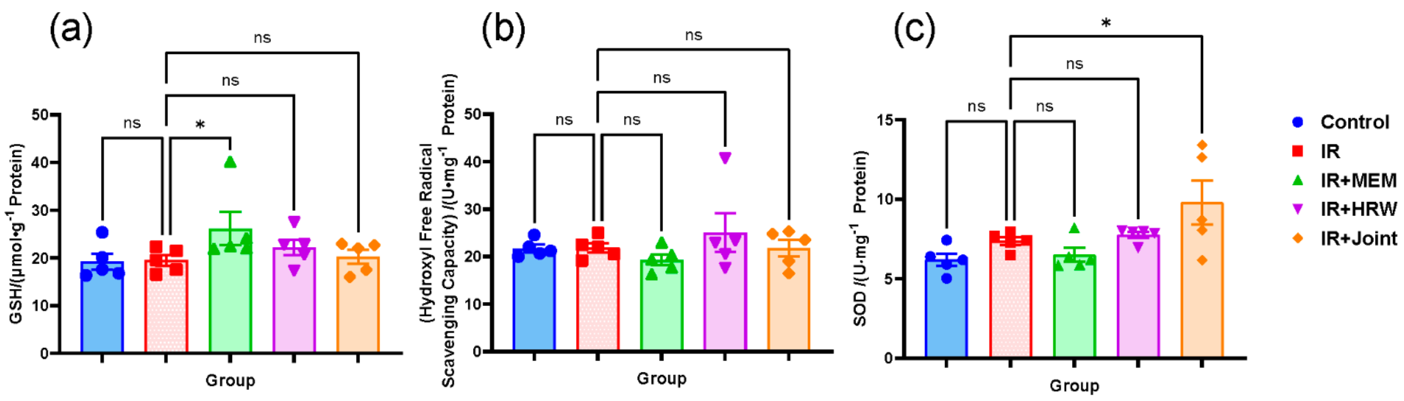

Figure S3. Brain tissue GSH, OH^{-}, and SOD levels. (a) GSH levels in brain tissue (n = 5). (b) Hydroxyl radical scavenging capacity (one unit of scavenging capacity is defined as the amount of protein required to reduce the concentration of hydrogen peroxide by 1 mmol/L in a 1-minute reaction at 37°C). (c) SOD

activity in brain tissue (n = 5). Statistical significance indicated as ns: not significant, *$p < 0.05$ compared with the IR group.

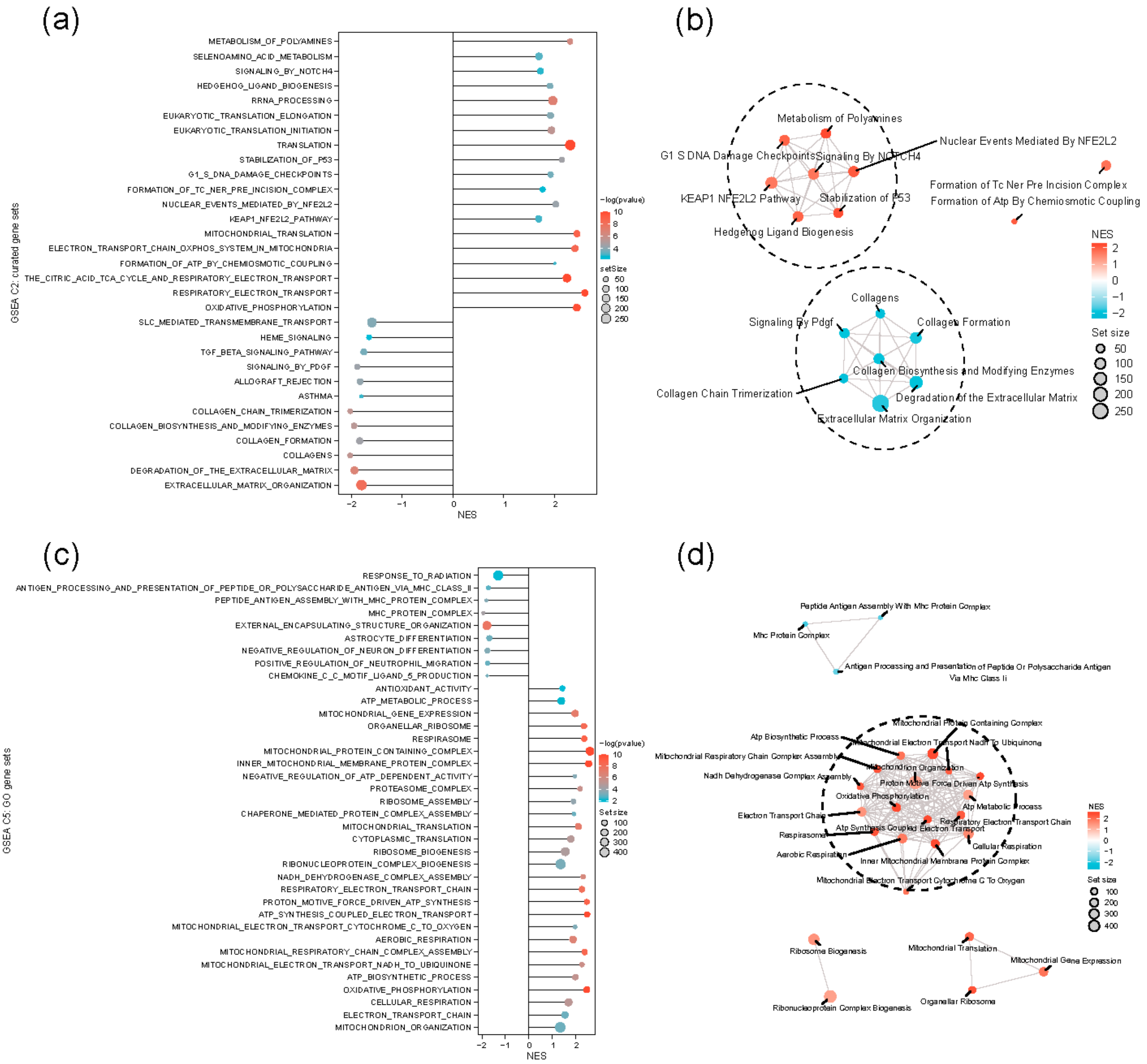

Figure S4. Gene Set Enrichment Analysis (GSEA).
(a) Lollipop plot of the C2: curated gene sets enrichment results.
(b) Cluster plot of the C2: curated gene sets enrichment results.
(c) Lollipop plot of the C5: GO gene sets enrichment results.
(d) Cluster plot of the C5: GO gene sets enrichment results.
